# Effects of Music Therapy on Postoperative Pain Perception in Patients Undergoing Abdominal Surgeries Under General Anesthesia: A Prospective Controlled Randomized Comparative Clinical Study

**DOI:** 10.7759/cureus.68019

**Published:** 2024-08-28

**Authors:** Subha Teresa J Vazhakalayil, Sanya Varma

**Affiliations:** 1 Anaesthesiology, DR. D.Y. Patil Medical College, Hospital and Research Centre, DR. D.Y. Patil Vidyapeeth University, Pune, IND

**Keywords:** lower abdominal surgeries, cortisol, general anaesthesia, postoperative pain, music therapy

## Abstract

Background

Music therapy has been shown to reduce the need for sedation and analgesics, as well as lower plasma cortisol and epinephrine levels in patients undergoing regional anesthesia. This study evaluates the efficacy of perioperative music therapy in reducing pain perception and its impact on cortisol levels.

Materials and methods

This prospective randomized control trial was conducted at the tertiary care center in Western Maharashtra. Sixty adult patients (males/females) undergoing elective lower abdomen surgery were included and randomized equally into two groups to listen to music using headphones at a preselected volume (Group M) or to use only headphones without any music played (Group C) for 2 hours in the preoperative, intraoperative, and postoperative periods. Demographic information, anthropometric (height, weight), and biochemical (serum cortisol) measurements were performed. American Society of Anesthesiologists (ASA) grades 1 and 2, and patients aged 18 to 65 years were included. Pain perception was assessed using the Visual Analog Scale (VAS).

Results

The demographic data, including mean age, BMI, ASA status, average duration of anesthesia, and average duration of surgery, were comparable between the two groups. Group M showed improved control of systolic blood pressure (SBP) at 10- and 30-minute post-induction of general anesthesia and had a significantly lower VAS score (p < 0.05). Additionally, patient satisfaction was higher in Group M (81.4%) compared to Group C (51.4%) with a p-value of < 0.05. Intraoperative awareness was non-significant between the groups.

Conclusion

Music therapy is a safe, cost-effective, and efficacious method for reducing pain perception and can be used in conjunction with other treatments for postoperative pain management.

## Introduction

Pain is a subjective experience influenced by physiological, psychological, and emotional processes [1]. It is often indicated by increases in systolic pressure, pulse, plasma cortisol levels, and stress responses [2]. Health professionals are particularly concerned about postoperative pain, which can vary widely in intensity and is sometimes inadequately managed [3,4]. Inadequate pain relief often results from poor assessment, misreporting, and patient-related factors, with opioids frequently failing to provide sufficient relief [4]. Poorly managed pain can lead to impaired sleep, and physical function, and can contribute to anxiety and depression, ultimately compromising the quality of life [5,6].

Both pharmacological and non-pharmacological interventions are necessary for effective postoperative pain management. Alternative methods, such as music therapy, can reduce analgesic dosage and frequency, thus minimizing drug-related side effects. Music has been shown to influence both physiological and psychological states by affecting subcortical brain areas, decreasing stress hormone responses, and inducing relaxation through modulation of the limbic system [7]. During surgery, music stimulation can decrease catecholamines, cortisol, and adrenocorticotropic hormone (ACTH) levels [8]. Studies have demonstrated that music therapy can reduce surgery-related anxiety, decrease the need for analgesics, and lower plasma cortisol and epinephrine levels in patients receiving regional anesthesia [9-12]. Evidence-based research supports the effectiveness of music therapy in hospital settings, showing positive impacts on recovery and reduced analgesic needs in patients under general anesthesia [13-15]. This study aims to evaluate the role of perioperative music therapy on postoperative analgesia and on the surgical stress hormone response of patients undergoing lower abdominal surgeries.

## Materials and methods

Study design and setting

The current study is a prospective, double-blinded randomized controlled trial to evaluate the effects of perioperative music therapy on postoperative analgesia and cortisol response. It was conducted in the Department of Anesthesiology and Critical Care at Dr. D.Y. Patil Medical College, Hospital & Research Centre, Pune, India, over four months. The study commenced following the approval of the Institutional Ethics Committee (I.E.S.C/83/2023).

Participants

The study included patients who met the following criteria: those classified as American Society of Anesthesiologists (ASA) grade 1 or 2, aged between 18 and 65 years, and both male and female participants [16]. The ASA physical status classification system was used to assess patients' physiological status and predict operative risk. Exclusion criteria were as follows: patients with ASA physical status of 3 or higher, those who refused to consent to the study, individuals younger than 18 or older than 65 years, and patients with psychological disorders or those taking antidepressant or antianxiety medications. 

Method of randomization

Simple randomization was employed to allocate participants into different groups. This method ensures that each participant had an equal chance of being assigned to either the control group or the music therapy group, thereby minimizing selection bias.

Intervention

In Group M, patients received music therapy by listening to music through headphones at a preselected volume for 2 hours in the preoperative, intraoperative, and postoperative periods. This intervention was designed to assess the impact of music on pain perception and stress levels. In Group C, patients used only headphones without any music played, serving as the control group. This setup allowed a comparison between the effects of music therapy and a placebo condition.

Measurements and data collection

We calculated the total sample size (n) as 60 (30 in each group). Sixty patients posted for lower abdominal surgeries electively requiring general anesthesia were included. Using a random allocation number, the population was divided into Group C (Control, n=30), which consisted of patients who received headphones without music. This group served as the control to compare the effects of music therapy. Group M (Music Therapy, n=30) consisted of patients who listened to non-lyrical music through headphones at a preselected volume for 2 hours in the preoperative, intraoperative, and postoperative periods. The music had a tempo of 60-80 beats per minute to avoid any potential increase in heart rate due to rhythmic entrainment.

Demographic data, including mean age, BMI, ASA grade, and the average duration of anesthesia and surgery, were systematically recorded and compared between the two groups. Pain perception was evaluated using the Visual Analog Scale (VAS) score, a standardized tool for assessing pain levels among patients. The VAS is a simple and effective tool for measuring pain intensity and other subjective experiences. It consists of a 10-centimeter line with endpoints labeled "No Pain" and "Worst Possible Pain." Patients mark a point on the line that represents their current pain level, with the distance from the "No Pain" end measured in millimeters to obtain a numerical score. This score, ranging from 0 to 100 mm, provides a quantitative measure of pain severity, with higher values indicating greater pain. The VAS is widely used in clinical practice and research for its ease of use and ability to quickly capture and track pain levels [17].

Blood samples were collected at two critical time points: after anesthetic induction and at the end of the surgery. The samples were transferred into sterile bottles within 45 minutes of collection and sent to the hospital's biochemistry laboratory for analysis. Cortisol levels were measured using a commercially available colorimetric enzyme-linked immunosorbent assay (ELISA) kit. All samples from each patient were analyzed in the same assay batch to ensure consistency.

Postoperatively, the study monitored the need for analgesics. The time until the first request for analgesics was recorded to evaluate the effectiveness of music therapy. Intravenous paracetamol (15 mg/kg) was administered as rescue analgesia for managing postoperative pain.

Outcome measures

Systolic blood pressure (SBP) was measured at several time points: baseline, premedication, after drug administration, and at 1, 3, 5, 10, 20, 30, 50, 70, 90, and 120 minutes post-induction. Heart rate (BPM) was recorded at similar intervals to monitor cardiovascular responses. Pain perception was assessed using the VAS at 30 minutes, 1 hour, 2 hours, 3 hours, and 4 hours post-induction. Cortisol levels were measured to evaluate stress response at induction and at the end of surgery.

Statistical analysis

SPSS Inc. Released 2007. SPSS for Windows, Version 16.0. Chicago, SPSS Inc. was used for data analysis. Data were analyzed using unpaired t-tests with a 95% confidence interval. A p-value of <0.05 was considered statistically significant. This analysis compared systolic blood pressure, heart rate, VAS scores, and cortisol levels between the two groups to determine the effectiveness of music therapy in reducing pain perception and its effects on physiological parameters.

## Results

The demographic parameters of the study participants are summarized for both Group M (Music Therapy group) and Group C (Control group) in Table 1. The mean age for Group M was 49.7±12.48 years, while for Group C, it was 50.3±13.33 years, indicating similar age distributions between the two groups. The Body Mass Index (BMI) was slightly higher in Group M (22.5±2.01) compared to Group C (21.8±1.95).

**Table 1:** Comparison of demographic and surgical data The mean age, BMI (basal metabolic rate), ASA (American Society of Anesthesiologists) grade and average duration of anesthesia and surgery did not show differences that were statistically significant. P<0.05- significant

Demographic		Group M	Group C	p-value
Mean age (in years)		49.7±12.48	50.3±13.33	0.86
BMI		22.5±2.01	21.8±1.95	0.176
ASA	ASA 1	16	14	-
	ASA 2	14	16	-
Average duration of anesthesia in mins		75.2±35.6	81.45±30.14	0.23
Average duration of surgery in mins		94.54±37.45	98.4±33.41	0.67

In terms of the American Society of Anesthesiologists (ASA) physical status classification, Group M had 16 (53%) participants classified as ASA 1 and 14 (47%) as ASA 2. Group C had 14 (47%) participants classified as ASA 1 and 16 (43%) as ASA 2, showing a balanced distribution of physical status between the groups.

The average duration of anesthesia was slightly shorter in Group M, with an average of 75.2 ± 35.6 minutes, compared to Group C, which had an average duration of 81.45 ± 30.14 minutes. Similarly, the average duration of surgery was 94.54 ± 37.45 minutes for Group M and 98.4 ± 33.41 minutes for Group C, indicating comparable surgical times between the two groups.

The comparison of systolic blood pressure between Group C and Group M at various time points shows several significant and non-significant differences (Table 2). At baseline, Group C had a mean systolic blood pressure of 116.42±10.6 mmHg, while Group M had 113.13±9.04 mmHg, with no statistically significant difference (p=0.264). Similar non-significant differences were observed premedication, after drug administration, and at 1, 3, and 5 minutes after drug administration. However, at the 5-minute mark, Group M's systolic blood pressure significantly decreased to 109.52±10.21 mmHg compared to Group C's 118.61±9.95 mmHg (p<0.05). This trend of significantly lower systolic blood pressure in Group M continued at the 10, 20, and 30-minute marks with p-values all less than 0.05. However, at 50, 70, 90, and 120 minutes, the differences between the groups were not statistically significant, indicating that the initial significant reductions in Group M's systolic blood pressure were not maintained throughout the monitoring period. These findings suggest that while there were early significant reductions in systolic blood pressure in Group M, these differences leveled out over time.

**Table 2:** Comparison of changes in systolic blood pressure An unpaired t-test with a 95% confidence interval was used for comparison. P<0.05 is considered significant.

Systolic Blood Pressure	Group M	Group C	p-value
Baseline	113.13±9.04	116.42±10.6	0.264
Premed	113.22±9.87	114±10.43	0.312
After Drug	110.1±9.70	110.84±10.95	0.612
After Drug 1 Min	112.69±9.49	113.66±10.17	0.233
After Drug 3 Min	113.3±10.1	114.96±9.91	0.455
After Drug 5 Min	111.82±10.18	112.92±10.37	0.264
5 Minutes	109.52±10.21	118.61±9.95	<0.05
10 Minutes	104.89±9.56	113.07±10.12	<0.05
20 Minutes	102.54±9.65	111.69±9.58	<0.05
30 Minutes	102.01±9.45	109.07±9.31	<0.05
50 Minutes	102.04±8.45	101.53±8.71	0.758
70 Minutes	102.04±8.15	101.73±7.21	0.545
90 Minutes	101.15±7.56	100.73±7.26	0.747
120 Minutes	101.94±6.99	101.5±6.80	0.655

Table 3 compares heart rates between two groups, Group C and Group M, at various time points. The analysis includes baseline measurements, premedication, and several intervals post-drug administration up to 120 minutes. The p-values were calculated using an independent samples t-test to determine if there were significant differences in heart rates between the two groups at each time point. The p-values at all time points exceed the threshold for statistical significance (p < 0.05), indicating that there are no statistically significant differences in heart rates between Group C and Group M. Overall, the results imply that the intervention or treatment administered did not produce significant differences in heart rate between the two groups at any measured time point. 

**Table 3:** Comparison of changes in heart rate An unpaired t-test to compare with a 95% confidence interval (2 sided). P<0.05 is considered significant.

Heart Rate (Bpm)	Group M	Group C	p-value
Baseline	86.59±15.71	85.98±11.11	0.863
Premed	82.76±9.81	81.92±9.21	0.734
After Drug	76.95±7.91	77.46±8.41	0.81
After Drug 1 Min	81.64±8.92	82.36±9.09	0.758
After Drug 3 Min	84.91±8.85	84.13±9.56	0.744
After Drug 5 Min	81.54±9.95	82.82±9.65	0.615
5 Minutes	85.59±15.91	81.01±11.19	0.202
10 Minutes	85.81±11.51	82.01±11.19	0.2
20 Minutes	85.01±11.05	79.78±10.71	0.068
30 Minutes	80.51±11.51	78.58±9.97	0.49
50 Minutes	79.19±11.58	79.11±8.11	0.975
70 Minutes	71.91±10.87	71.98±7.97	0.977
90 Minutes	71.98±8.91	71.18±7.57	0.709
120 Minutes	75.18±8.10	75.39±7.70	0.918

Table 4 provides a comparison of Visual Analog Scale (VAS) scores between Group C and Group M at various time intervals post-treatment: 30 minutes, 1 hour, 2 hours, 3 hours, and 4 hours. At 30 minutes, the VAS score was 3.75 ± 0.95 for Group C and 3.65 ± 0.74 for Group M, with a p-value of 0.651. At 1 hour, the score was 4.21 ± 0.96 for Group C and 4.11 ± 0.81 for Group M, with a p-value of 0.664. At 2 hours, the score was 4.59 ± 0.86 for Group C and 4.38 ± 0.89 for Group M, with a p-value of 0.357. At 3 hours, the score was 4.96 ± 1.15 for Group C and 4.73 ± 1.11 for Group M, with a p-value of 0.434. At 4 hours, the score was 5.54 ± 1.01 for Group C and 5.07 ± 1.09 for Group M, with a p-value of 0.089. There was no statistically significant difference in VAS scores between Group C and Group M at any of these time intervals.

**Table 4:** Comparison of changes in VAS Score

Time Interval	VAS Score (Group M)	VAS Score (Group C)	p-value
30 Mins	3.65 ± 0.74	3.75 ± 0.95	0.651
1 Hour	4.11 ± 0.81	4.21 ± 0.96	0.664
2 Hour	4.38 ± 0.89	4.59 ± 0.86	0.357
3 Hour	4.73 ± 1.11	4.96 ± 1.15	0.434
4 Hour	5.07 ± 1.09	5.54 ± 1.01	0.089

Table 5 presents cortisol scores for Group C and Group M at two key time intervals: during induction and at the end of surgery. At induction, Group C had a mean cortisol score of 31.42 ± 14.2, while Group M had a mean score of 28.2 ± 13.7, with a p-value of 0.375. At the end of the surgery, the mean cortisol score of Group C was 32.9 ± 11.2, compared to 29.4 ± 10.6 for Group M, with a p-value of 0.219 indicating no significant difference.

**Table 5:** Comparison of changes in cortisol levels before and after surgery

Time Interval	Cortisol Score (Group M)	Cortisol Score (Group C)	p-value
Induction	28.2 ± 13.7	31.42 ± 14.2	0.375
End of Surgery	29.4 ± 10.6	32.9 ± 11.2	0.219

## Discussion

The present study observed lower scores in the music group, though these differences were not statistically significant. Palmer et al. evaluated patient satisfaction using a five-item score and found no significant differences between groups when assessing each item individually [18]. Conversely, Dubios et al. reported a significant improvement in patient satisfaction with music as a general anesthetic during bronchoscopy (p=0.02) [19].

In terms of intraoperative hemodynamic stability, the current study found better outcomes in group C, particularly regarding variations in systolic blood pressure (SBP). Similar findings were reported by Turner et al. [20]. The study noted significant changes in SBP in the music therapy group at 5, 10, 20, and 30 minutes after anesthesia induction. However, no significant differences were observed between the groups concerning heart rate. This aligns with several studies on hemodynamic changes [21,22]. For instance, Jaber et al. reported significant reductions in heart rate (88±15 vs. 82±15 bpm, p <0.05) and SBP (137±17 vs. 128±14 mmHg, p <0.05) [21], while similar results were observed in studies by Mary Kay Williams and Steelman VM et al. [22 23]. Variations in results may be influenced by the selection and type of music played, as well as the timing of its introduction. Binns-Turner et al. noted the beneficial effects of music played in the postoperative period, with potential changes in blood pressure attributed to neurohormonal modulation responses [20].

This study noted lower cortisol levels in group M at both induction and the end of surgery, although the differences were not statistically significant. Wang et al. and Migneault et al. also measured intraoperative plasma cortisol and found no significant differences between groups [24,25]. Despite different methods for assessing plasma cortisol, their findings align with the results of this study.

Additionally, the study found lower Visual Analog Scale (VAS) scores in group M, but these differences were not significant. Binns-Turner et al. measured VAS scores both preoperatively and at discharge from the post-anesthetic care unit, revealing that the intervention group had significantly lower scores at discharge (41.5±30.2 vs. 64.9±20.9; p = 0.007) [20]. Similarly, Jayaraman et al. investigated the analgesic effects of music therapy in 111 patients undergoing laparoscopic gallstone surgery and found a significant increase in pain intensity in the control group compared to the intervention group [26].

Limitations of the study: In our study, we couldn't demonstrate a statistically significant difference in the stress hormone response as measured by serum cortisol levels between the two groups. Measurement and analysis of serum cortisol levels was a time-consuming and tedious process. The study duration of four months was too long.

## Conclusions

Music therapy is a safe, cheap, and effective method for reducing pain perception and can be used with other methods in treating postoperative pain. The present study evaluated the effects of perioperative music therapy on postoperative pain perception and stress hormone response as measured by cortisol levels. The results showed a statistically significant difference in the pain scores measured by VAS among the study and control groups. There was a statistically significant prolonged duration of analgesia in the study group. However, there was no statistically significant difference found in the cortisol levels in both groups. Stress as measured by cortisol was not significant. Music therapy has been understood to be beneficial in managing even surgical patients undergoing operations under general anesthesia. By utilizing this simple, noninvasive, nonpharmacological, and cost-effective technique, patients are improved satisfactorily. Most studies on this aspect, some of which have considerable scientific power, suggest effective implementation of the treatment protocol during the perioperative period. 
